# p53, a novel regulator of lipid metabolism pathways

**DOI:** 10.1186/1753-6561-6-S3-P61

**Published:** 2012-06-01

**Authors:** Ido Goldstein, Osnat Ezra, Noa Rivlin, Alina Molchadsky, Shalom Madar, Naomi Goldfinger, Varda Rotter

**Affiliations:** 1 Department of Molecular Cell Biology, The Weizmann Institute of Science, Rehovot, Israel

## Background

In this study we aimed at characterizing the regulation of hepatic metabolic pathways by the p53 transcription factor.

## Materials and methods

Analysis of gene expression following alteration of p53 status in several human- and mouse-derived cells using microarray analysis, quantitative real-time PCR, chromation immunoprecipitation and reporter gene assays. A functional assay was performed to determine lipid transfer activity.

## Results

We identified a novel role for the p53 protein in regulating lipid and lipoprotein metabolism, a process not yet conceived as related to p53, which is known mainly in its tumor suppressive functions. We revealed a group of 341 genes whose expression was induced by p53 in the liver-derived cell line HepG2. Twenty of these genes encode proteins involved in many aspects of lipid homeostasis. The mode of regulation of three representative genes (*plfp*, *abca12* and c*el*) was further characterized. In addition to HepG2, the genes were induced following activation of p53 in human primary hepatic cells isolated from liver donors, p53-dependent regulation of these genes was evident in other cell types namely Hep3B cells and mouse hepatocytes. Furthermore, p53 was found to bind to the genes’ promoters in designated p53 responsive elements and thereby increase its transcription. Importantly, p53 augmented the activity of secreted PLTP, which plays a major role in lipoprotein biology and atherosclerosis pathology**.**

## Conclusions

These findings expose another facet of p53 functions unrelated to tumor suppression and render it a novel regulator of hepatic lipid metabolism and consequently of systemic lipid homeostasis and atherosclerosis development.

